# Development and Validation of SEC-UV/HRMS Procedure for Simultaneous Determination of BSA and Its Association Products

**DOI:** 10.3390/molecules31061001

**Published:** 2026-03-16

**Authors:** Blaž Hodnik, Žiga Čamič, Matevž Pompe

**Affiliations:** 1 Krka d.d., R&D, Šmarješka Cesta 6, 8001 Novo Mesto, Slovenia; blaz.hodnik@krka.biz (B.H.); ziga.camic@krka.biz (Ž.Č.); 2 Faculty of Chemistry and Chemical Technology, University of Ljubljana, Večna Pot 113, 1000 Ljubljana, Slovenia

**Keywords:** size exclusion chromatography, proteins, high-resolution mass spectrometry

## Abstract

Monitoring peptide and protein self-association is essential for understanding biological function, formulation stability, and aggregation mechanisms. While size-exclusion chromatography (SEC) is routinely used to quantify protein-size variants under native conditions, its hyphenation to high-resolution mass spectrometry (HRMS) for simultaneous structural characterization remains limited. Here, we report the development and validation of a robust SEC-UV/HRMS method optimized for native-like analysis of bovine serum albumin (BSA) monomers and higher-order oligomers using standard-flow electrospray ionization. Systematic evaluation of source parameters, mobile-phase composition, and chromatographic conditions enabled retention of native BSA structure, minimized in-source unfolding, and enhanced MS sensitivity, allowing detection of oligomers up to the heptamer. A short, narrow-bore 200 Å UHPLC SEC separation column was used. Low-flow separations (~0.05 mL/min) enabled efficient ionization and 10 min run times. An accelerated 60 °C stress-testing protocol demonstrated that SEC-MS can semi-quantitatively monitor oligomerization dynamics, complementing UV-based quantification and revealing transient species not resolved by UV alone. The method showed acceptable linearity, precision, and sample stability, and comparison with SEC-RALS/LALS confirmed molecular-weight trends across aggregation states. Overall, the developed SEC-UV/HRMS workflow provides a rapid, sensitive, and widely accessible approach for UV-based quantification of monomer- and HRMS-based characterizing protein aggregation in research and quality control in pharmaceutical laboratories.

## 1. Introduction

Various larger peptides and proteins are nowadays increasingly important in numerous fields—from therapeutic biopharmaceutical development and protein-based biomaterials to studies of protein self-association, oligomerization, and aggregation. Monitoring not only the monomeric species, but also their higher order assemblies (dimers, trimers, oligomers, aggregates), is critically important because mentioned assemblies can impact biological activity, stability, immunogenicity, or formulation behavior (for instance, in bio-pharmaceutical drugs) and also for basic science in studying self-association mechanisms [1,2,3,4].

Traditional analytical methods for peptides and proteins (e.g., reversed-phase liquid chromatography coupled to MS) are very powerful for sequence identification, post-translational modification mapping, and quantification of monomeric species [5,6]. All these techniques usually lead to denaturation of the system and are therefore not suitable for characterization of monomers and their associates in native form. Therefore, when it comes to characterizing size-variants or assemblies (e.g., oligomeric or aggregated forms) of polypeptides, some optical analytical techniques like various laser and X-ray techniques are more suitable. The topic was described in extensive review article [7]. However, size-exclusion chromatography (SEC) represents a standard technique in biopharmaceutical industry for assessing aggregate content and size variants under near-native or native-like conditions [8,9,10]. Part of the standard analytical procedure for the characterization of the formulation in biopharmaceutical field is assay analysis together with the quantification of associates. On one side, the optical techniques provide fast results, but they are falling behind in quantitative analysis compared with SEC [11].

Nevertheless, applying SEC to polypeptides poses several challenges. Support materials and packing dimensions designed for large proteins may lack adequate resolution for smaller molecular weights (<10 kDa) or may suffer from secondary interactions (hydrophobic or ionic) that distort retention behavior [10,12,13,14]. Secondly, when the goal is not only to separate size variants but also to identify the species (e.g., determine exact molecular mass, stoichiometry of oligomers, or modifications), SEC alone (with UV/RI/MALS detectors) is insufficient [15,16]. Coupling to mass spectrometry (MS) adds significant analytical power, enabling accurate mass measurement, detection of heterogeneity (modifications, oligomer variations), and quantification via extracted-ion chromatograms (EICs) or similar approaches [17,18,19].

Despite the conceptual attractiveness of SEC for proteins and aggregate analysis, coupling SEC directly to high-resolution mass spectrometry (HRMS) remains relatively underexplored [18,19,20]. Several studies on proteins and antibody–drug conjugates show the promise of SEC-HRMS for detailed molecular characterization: for example, the integration of denaturing or native SEC with high-resolution MS to probe higher-order structures and modifications of monoclonal antibodies [21,22,23,24]. In polymer chemistry, the hyphenation of SEC to electrospray ionization mass spectrometry (ESI-MS) has also been demonstrated as a powerful tool for determining distributions and end-group chemistries in synthetic macromolecules [25]. These precedents both emphasize the potential and highlight the methodological complexity of coupling a size-based separation to a high-sensitivity structural detector.

The analytical determination of proteins and their aggregation states benefit from such a hyphenated approach for several reasons. Firstly, SEC resolves species primarily by size, enabling separation of monomeric, dimeric, or higher-order peptide assemblies without relying on sequence or charge differences [10]. Secondly, the subsequent HRMS detection provides accurate mass measurement, isotopic pattern insight, and the ability to identify individual polypeptide species and aggregates, thus bridging the gap between separation and structural characterization [18]. Thirdly, in complex biological or synthetic polypeptide mixtures, the combination enables both qualitative discrimination and quantitative estimation of size-based heterogeneity [23,26].

However, several analytical challenges must be addressed to enable a robust SEC-HRMS procedure for polypeptides. On the one hand, we need to achieve adequate resolution in the SEC dimension and minimal sample dilution (a common limitation of SEC) by selecting a mobile phase compatible with ESI and high-resolution mass spectrometry. On the other hand, we need to optimize MS parameters for multiple charge states and possible aggregate formation. Indeed, older work highlighted that even HPSEC for polypeptides required attention to column packing, flow-rate selection, sample volume, and interaction minimization.

The aim of this study was to demonstrate that the limitations of standard-flow electrospray ionization can be largely overcome by systematic optimization of the solvent system used for separation and of the source conditions used for ionization. To obtain compatibility between near-native SEC separation and MS detection, the effects of pH, organic modifiers, and lowered buffer concentration on ionization performance were studied by using direct infusion to a mass spectrometer. Further screening of chromatographic conditions using modern UP-SEC columns was done to achieve improvements in sensitivity by enabling separation at flows that are similar to direct infusion MS but are still compatible with commercially available UHPLC pumps used for standard flow separations. The developed analytical procedure was validated. The presented detection system was compared with alternative commonly used detectors in SEC analysis; that is, UV and refractive index (RI) and light scattering (RALS/LALS) detectors. Bovine serum albumin was used as a model system for method development and optimization, and the applicability of the method was demonstrated by the analysis of stressed samples of BSA to monitor its aggregation propensity in different media.

## 2. Results

### 2.1. Development of SEC-UV/MS Method for Determining Monomeric BSA

Initially, an SEC method was developed to monitor the concentration of monomeric bovine serum albumin in aqueous solution. The method needed to be able to separate monomeric analyte from higher and lower molecular weight species while enabling determination of molecular weight of the main species using MS. To establish a starting point from which the effect of individual parameters could be studied, two commercially available size-exclusion columns were tested for suitability; that is, Waters UPLC Protein BEH (Milford, MA, USA), 125 Å, 300 × 4.6 mm, 1.7 µm, and Agilent AdvanceBio SEC (Santa Clara, CA, USA), 120 Å, 300 × 4.6 mm, 1.9 µm.

Both columns were chosen based on their size resolving range, which is dictated by pore size (measured in Å) and ranged up to 80 kDa according to the manufacturer’s specifications. As the molecular weight of BSA is approximately 66 kDa, both columns were expected to separate monomer and oligomer species within a sample of stressed BSA. Ammonium acetate was chosen as a mobile phase buffer system according to the evaluation of different mobile phase additives as reported in the literature [27,28]. The mobile phase was comprised of 50 mM ammonium acetate in purified water adjusted to physiological pH (7.4). A sample of BSA was prepared in ammonium acetate and stressed in a dry heating block at 50 °C for a period of 2 weeks to induce aggregation. The solutions were analyzed according to the chromatographic and source conditions as stated in the experimental section. Initial run time was set to 30 min due to prolonged retention of the solvent front, which is caused by the lower flow rates needed for coupling to MS.

**Figure 1:**
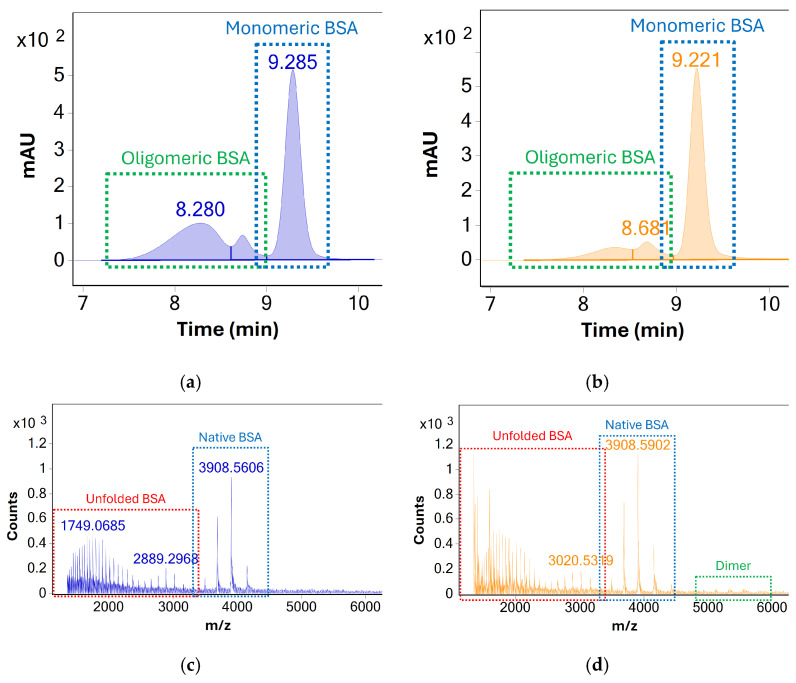
UV (280 nm) chromatograms of the stressed BSA samples acquired using the (**a**) Waters UPLC Protein BEH, 125 Å, 300 × 4.6 mm, 1.7 µm column and (**b**) Agilent AdvanceBio SEC, 120 Å, 300 × 4.6 mm, 1.9 µm column. MS spectra averaged across all chromatographic peaks, acquired using the (**c**) UPLC Protein BEH (**d**) AdvanceBio are shown below. The higher charge states of monomeric BSA which indicate analyte denaturation are labelled in red. The response for dimeric BSA (as a consequence of both in situ and in source oligomerization) is labelled green. The latter is significantly more pronounced when using the Agilent column.

Column performance was evaluated based on UV and MS response—see Figure 1. In the case of both columns, monomeric BSA was well separated from lower and higher molecular weight species. Two distinct peaks attributed to higher molecular weight species (oligomers and/or aggregates) were observed but could not be unambiguously identified due to poor MS response. For monomeric BSA, the AdvanceBio SEC column produced higher UV peak areas, increased UV signal-to-noise ratios, and superior MS response despite indicating an overall lower abundance of higher molecular weight species. Additionally, the presence of dimeric BSA could be confirmed using the AdvanceBio SEC column based on the MS response of the peak at Rt 8.7 min, whereas no such MS response could be detected using the Protein BEH column.

Based on these findings, the AdvanceBio SEC column was identified as the superior option for monitoring the concentration of monomeric BSA during stress testing. The existing method was identified as a promising starting point for the development of an SEC-UV-MS method, which could identify and monitor both monomeric BSA and higher molecular weight species simultaneously. While UV detection under existing conditions was sufficient for analysis of BSA and its association products, the method showed several limitations in terms of mass analysis (see Figure 1c,d). The MS response for monomeric BSA showed a very high degree of protein unfolding during analysis, as evidenced by the presence of highly charged BSA species visible between *m*/*z* 1300 and *m*/*z* 3000. At the same time, only limited MS response of oligomeric species was visible, limiting the identification of oligomers beyond dimeric BSA.

Several areas of interest were identified, which should be investigated in order to overcome these limitations. Firstly, source conditions should be optimized to decrease protein unfolding during ionization and mass analysis, allowing for detection of various BSA species in their native form. Secondly, the effect of mobile phase concentration, pH, and solvent composition should be investigated to optimize vaporization and to identify any potential causes of on-column denaturation. Additionally, chromatographic performance should be improved in order to meet the criteria for a stability indicating method that is used for high-throughput stress testing of protein samples. Larger column pore sizes should be investigated in order to increase chromatographic separation of oligomer species and higher aggregates. Column diameter should also be decreased to enable separation at lower flow rates and shorter run times, which are needed for native MS analysis and for shorter sampling times.

Finally, validation parameters such as repeatability and linearity should be investigated in order to evaluate the reliability of the method for long-term use in regulatory and research environments. The results as obtained by the developed method should be compared to an orthogonal detection technique, i.e., MALS detection. The investigation of each parameter is described below with both experimental design and result discussion included.

### 2.2. Optimization of Source Parameters

To ensure native conditions throughout ionization and mass analysis and optimal sensitivity, the source conditions were optimized systematically according to two criteria: the ratio between higher and lower charge states (i.e., charge state distribution, which is indicative of the ratio between folded and unfolded protein) and signal intensity. The parameters were optimized via flow injection analysis of a 100 µg/mL solution of BSA into a 0.2 mL/min flow of mobile phase in order to increase throughput and replicate the conditions in the ion source when used in tandem with SEC separation.

#### 2.2.1. Effect of Gas Temperature

When comparing the spectra of BSA at different gas temperatures, a clear correlation between the temperature and charge-state distribution was observed. As a sheath gas temperature str increased, the charge-state distribution shifts towards higher charge states, indicating that heat-induced unfolding of BSA occurs at higher gas temperatures. In terms of sensitivity, a reverse correlation between sheath gas temperature and BSA signal intensity was observed, indicating that loss of sensitivity may be a necessary trade-off when ensuring native ionization conditions. As the loss of intensity can most likely be attributed to the inability of the source to vaporize the mobile phase, we identified mobile phase-flow rate as a critical parameter for optimization during chromatographic method development. In this area, the use of modern ultra-high-pressure columns that can maintain sufficient pressure at lower flow rates was identified as a possible alternative to standard high-pressure columns.

#### 2.2.2. Effect of Gas Flow and Source Voltages

In contrast to gas temperature, the flow rate of nebulization, sheath, and drying gases had a relatively minor effect on protein unfolding—increasing or decreasing the individual flow rates produced only minor changes in charge-state distribution, indicating that any potential turbulence and shear forces that might arise from higher flow of gas do not impact the native structure of bovine serum albumin. Similarly, the effect of gas-flow rate on signal intensity seems to be limited as well. Despite any increase in sensitivity being limited, a positive correlation between gas flow and signal intensity was observed for all three gases used, especially for drying gas, which can largely be attributed to more efficient mobile-phase vaporization at higher gas-flow rates. Because the mobile phases used in native separations are generally highly aqueous, it is expected that higher flow rates will be preferred for most analytes.

**Figure 2:**
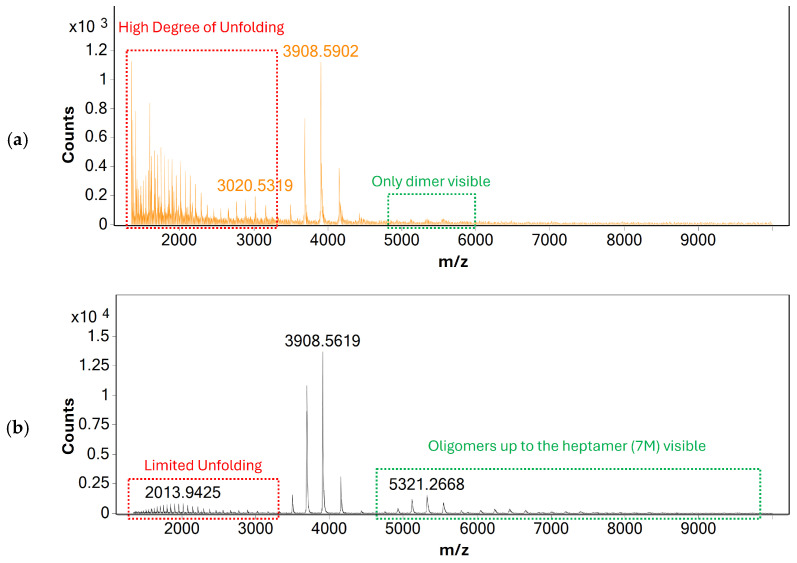
MS spectra of BSA acquired using direct infusion under (**a**) initial and (**b**) the optimized source conditions. The spectrum acquired using optimized source conditions shows only limited response for unfolded BSA, indicating that the native structure of BSA is largely retained. Additionally, oligomers of BSA up to the heptamer (7 M) are visible, indicating that non-covalent protein-protein interactions are retained even after ionization and transfer to the gas phase.

Alongside gas flow, source voltages were optimized to improve the sensitivity of mass analysis and to investigate their effects on protein unfolding. Similarly to gas-flow rate, no effect of source voltages on the charge-state distribution of BSA was observed, indicating that heat-inducted denaturation is the main cause for protein unfolding during mass analysis. Based on these findings, individual parameters such as capillary and nozzle voltages were optimized to produce the maximum response of monomeric BSA possible—see Figure 2.

### 2.3. Optimization of Chromatographic Separation

Following the optimization of mobile phase and source conditions, the method was transferred to a separation column, which was chosen based on the considerations listed above: firstly, a larger pore size was used that enables separation of a wider range of higher molecular weight species, and, secondly, a smaller inner diameter was chosen to enable analysis at lower flow rates and shorter run times. Additionally, the use of PEEK inner housing was introduced to limit any secondary interactions, which might arise due to the use of relatively low buffer concentration. The column chosen was the Agilent AdvanceBio SEC, 200 Å, 150 × 2.1 mm, 1.9 µm PK, which met all the criteria listed above. During method transfer, the flow rate of the mobile phase was lowered to 0.05 mL/min to increase ionization efficiency at lowered temperatures, and the run time was decreased to account for this. The concentration of ammonium acetate in the mobile phase was lowered from 50 mM to 20 mM to increase MS sensitivity and decrease maintenance costs. In place of pure water, the ammonium acetate was dissolved in a 20:80 (*v*/*v*) mixture of methanol and water to improve mobile phase volatility and ionization efficiency. The response of unfolded BSA and higher-molecular-weight species was monitored throughout mobile phase optimization to ensure that native conditions were retained.

**Figure 3:**
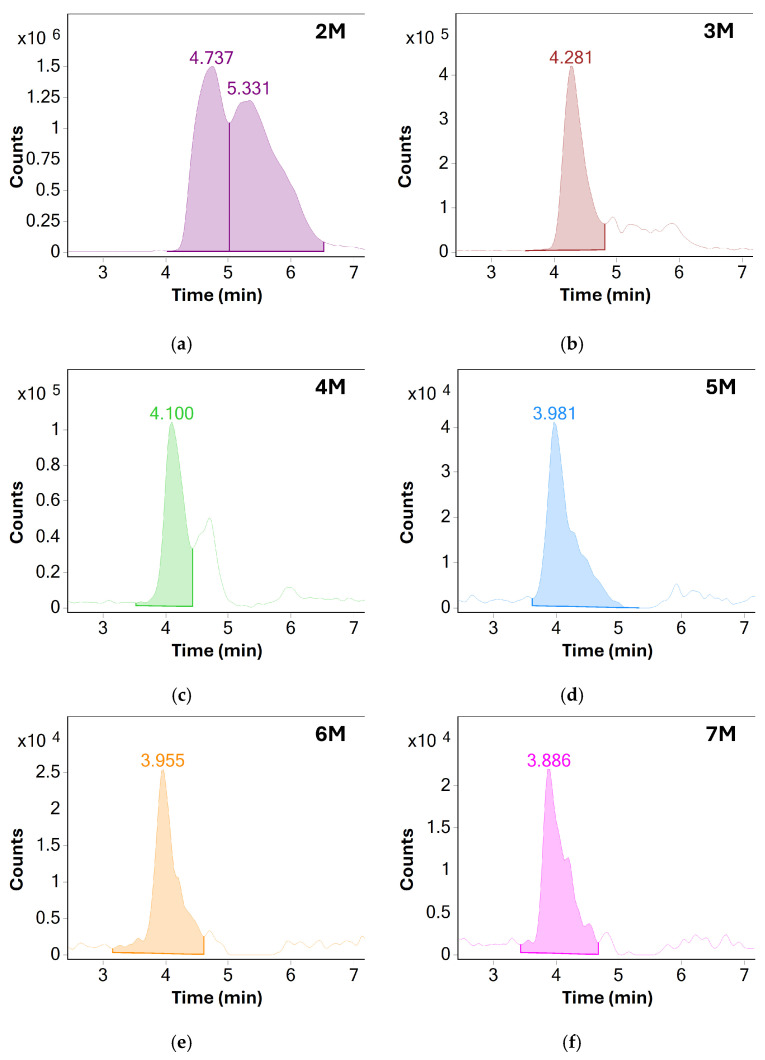
Extracted ion chromatograms (EIC) of various oligomeric species of BSA: (**a**) dimers, (**b**) trimers, (**c**) tetramers, (**d**) pentamers, (**e**) hexamers and (**f**) heptamers. Based on the measured S/N values, only oligomers up to the pentamer were chosen for monitoring in later experiments, additional method optimization may allow for monitoring of higher molecular weight species in the future.

When comparing the results before and after method optimization, overall sensitivity of MS detection was greatly increased, especially for higher molecular weight species. These could now be reliably detected using MS even in unstressed samples of BSA. Because of shorter column length, the separation between individual oligomeric species was reduced. However, a single distinct chromatographic peak for each species could now be produced by extracting their respective *m*/*z* responses, further showcasing the benefit of MS detection—see Figure 3.

Furthermore, an additional chromatographic peak could be detected using UV, which eluted slightly before the chromatographic peak of oligomeric BSA, indicating the presence of higher molecular weight aggregates. The peak did not produce a detectable MS response, most likely because of their very high neutral mass, which is outside the capabilities of the instrument used. The lack of MS response may also be due to the much wider molecular weight distribution, which in turn produces a much greater number of MS signals at lower intensity compared to the few high-intensity signals of oligomeric and monomeric species.

The transition to a shorter, smaller inner diameter column also increased the throughput of the method by reducing the run time from 30 min down to approximately 10 min, making it much more suitable for time-sensitive stress testing of samples. The reduced flow rate also limited mobile phase use, so analysis is now constrained by mobile phase stability rather than sample size or solvent availability. The use of only small amounts of LC-MS grade water, methanol, and ammonium acetate makes it highly economically viable, and because no specialized or modified MS instrumentation is needed, it is easily transferable to most research and development environments, even those not yet specialized in native mass spectrometry applications.

### 2.4. Stress Testing of BSA & Determination of Higher Molecular Weight Species

To showcase the applicability of the developed SEC-MS method, an accelerated stress-testing protocol was developed, which would enable monitoring BSA aggregation under varying conditions. Similar stress-testing protocols are widely used in various industries, especially in pharmaceutical research and development, where they are a powerful tool for predicting the stability of drug products and formulations. As the optimized method already allowed for rapid analysis at 10 min per sample, our aim was to develop an accelerated protocol, which could be performed in a matter of hours to allow for same-day stress testing and sample analysis.

Preliminary stress testing of the samples used for method development already showed that BSA undergoes detectable aggregation at 50 °C. However, the formation of aggregates was limited even after 14 days of stress testing. To further optimize the temperature of the stress testing, solutions of BSA were stress tested at 50 °C, 60 °C, and 70 °C for up to an hour and sampled at predetermined timepoints: after 5, 15, 30, and 60 min of stress testing.

**Figure 4:**
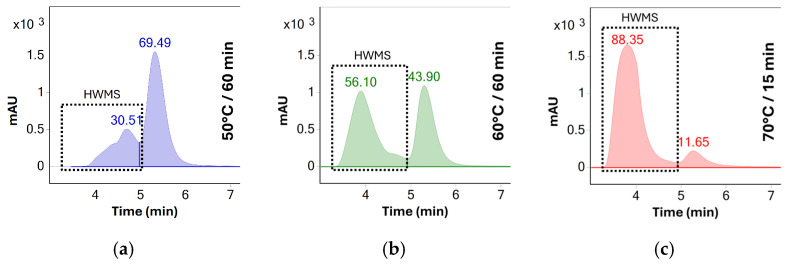
UV chromatograms (280 nm) of BSA solutions stressed at different temperatures: (**a**) 60 min at 50 °C, (**b**) 60 min at 60 °C, and (**c**) 15 min at 70 °C. The duration of stress testing at 70 °C was shortened due to the solution becoming opalescent after 30 min. The number above each chromatographic peak represents the relative abundance of the peak as determined by the area percent method.

Initial screening of different temperatures was performed using UV detection in order to determine the degree of aggregation after each time point. The chromatographic peaks of monomeric BSA and of higher molecular weight species (HMWS) were integrated, and the peak areas were determined. Due to limited separation between higher weight species at later time points, the peak areas of oligomers and aggregates were summed and reported as “higher molecular weight species” or HMWS. Relative concentrations of HMWS and monomeric BSA were calculated as percentages of total detected protein response—see Figure 4.

Results show no significant aggregation of the sample solutions stressed at 50 °C. The rate of aggregation observed indicated that a significant amount of aggregates would be formed only after several days of stress, making the stress testing protocol impractical for same-day analysis. In contrast, stress testing at 70 °C increased the percentage of aggregates from 30% to 88% after only 15 min of stress testing. Additionally, the solution became opalescent after 30 min of stress testing at this temperature, making it unusable for HPLC analysis due to the risk of damage to the system. As such, 60 °C was determined to be the ideal temperature for accelerated testing as no opalescence was observed throughout the 60 min testing period and the percentage of oligomeric species increased to approximately 56% after this period—see Table 1.

**Figure 5:**
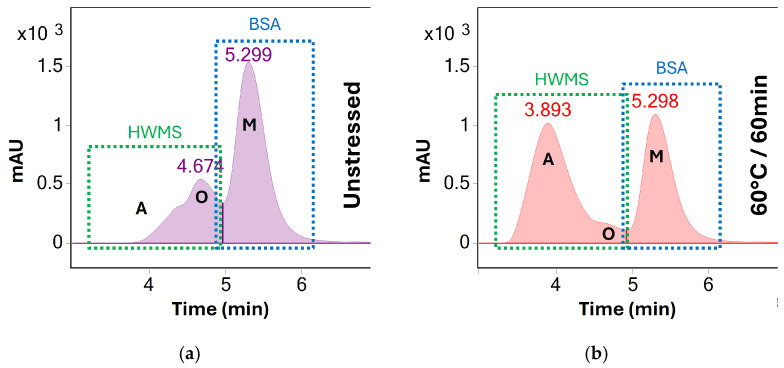
UV (280 nm) chromatograms of BSA (**a**) before stress testing and (**b**) after heating at 60 °C for 60 min. A clear increase of higher order aggregates (**A**, visible at 3.9 min) is observed as the concentration of oligomeric (**O**, 4.7 min) and monomeric BSA (**M**, 5.3 min) decreases. Unlike at 70 °C, all three peaks remain separated throughout stress testing at 60 °C. However, peaks A and O are summed and reported as HMWS for preliminary screening for the sake of consistency.

After UV screening, the stress testing procedure at 60 °C was repeated with MS detection. UV detection at 280 nm was used to quantify the total degree of oligomerization and aggregation of the area percentages as described above. However, oligomers and aggregates were reported as separate species where possible—see Figure 5.

For MS detection, the most abundant charge state for each species was determined and extracted ion chromatograms for the most abundant charge state were generated using a 50 Th extraction window. The peak area for each oligomeric species was determined via integration and reported either as absolute response or as relative response normalized to the area determined at the start of stress testing. Because standards for each non-covalently bound oligomer species are not available, absolute quantificationwas not possible. However, by comparing the absolute peak areas and normalizing them for each individual species, changes in species concentration could be monitored throughout the stress testing process.

**Figure 6:**
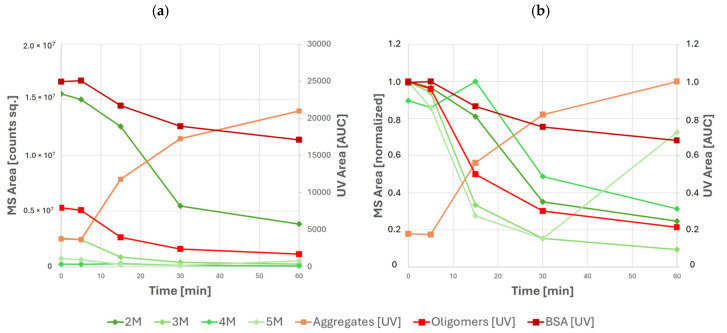
Abundances of various species of BSA and its high-molecular-weight species as measured by UV and MS during stress testing: (**a**) absolute peak areas and (**b**) relative peak areas normalized to the maximum of each individual species. UV data are shown in orange and red; MS data are shown in green.

This approach provided an additional insight into the oligomerization dynamics where UV detection could not discern between different oligomeric species due to coelution. The benefits of a more granular approach to oligomerization monitoring are visible in the results of stress testing at different temperatures—here, results of UV-based quantification indicate a steady decrease of all oligomeric species with time, whereas results of MS analysis clearly indicate that the abundance of tetrameric and pentameric BSA increases at various points of stress testing—see Figure 6.

Specifically, local maxima of tetramer and pentamer abundance are observed after 15 min and 60 min of stress testing, respectively. The use of MS-based detection also becomes increasingly important in later time points of stress testing, where the increased abundance of larger aggregates causes coelution of aggregate and oligomeric BSA species. In this case, oligomeric BSA can no longer be quantified using UV. However, the abundance of individual oligomeric species can still be monitored using MS. Nevertheless, mass analysis alone cannot be used for absolute quantification due to the differences between ionization efficiency, ion transmission, and detector response between species of varying molecular weight.

Ultimately, a combined approach utilizing both UV and MS detection is the most powerful. The use of UV allows for reliable quantifications on monomeric, oligomerized, and aggregated BSA by comparing their response to a standard solution of monomeric BSA using the external standard method. The use of MS in tandem with UV additionally allows for more granular monitoring of the abundance of individual HMWS and for the exact identification of each chromatographic peak based on its molecular weight.

### 2.5. Method Validation

To showcase the suitability of the method for use in more regulated environments, basic validation parameters such as linearity, repeatability, precision, and solution stability were determined. The methodology and acceptance criteria are stated in Section 3. The results of the method validation are presented below—see Table 2.

The method was shown to be linear for the UV detection of monomeric BSA from 0.02 mg/mL to 20 mg/mL and for the UV detection of oligomeric BSA from 0.2 mg/mL to 20 mg/mL. However, it must be mentioned that differences in relative responses of individual oligomers will cause the shift from linearity during the degradation study, since various oligomers will contribute to the overall signal for oligomer species. The full evaluation of linearity would be possible only by complete separation of all oligomers or presence of appropriate standards. Using UV detection, the relative standard deviations of the peak areas of monomeric and oligomeric BSA were well below 5.0% down to 0.2 mg/mL of total BSA content, meaning that the method was precise down to at least 1% of the working concentration—additionally, the RSD for monomeric BSA was nearly acceptable even at 0.02 mg/mL, meaning that the method is most likely precise even below 1% of the working concentration. In terms of repeatability, the method met the acceptance criteria for UV detection at all time points of stress testing, indicating that the developed stress testing protocol in tandem with UV detection can produce reliable and repeatable results despite very rapid sampling. The solution of BSA was shown to be stable even after 24 h of storage at 7 °C, as evaluated by the UV response of monomeric and oligomeric BSA.

When using MS detection of monomeric and oligomeric BSA, method precision at lower working concentrations was less ideal, with some relative standard deviations exceeding 15% at 0.02 and 0.02 mg/mL of total BSA. Nevertheless, at standard working concentrations of 20 mg/mL, the relative standard deviation of all detected oligomeric species was well below the acceptance criterion of 15%, as were the relative standard deviations at different points in stress testing. Similarly, the stability of the sample solution was shown to be acceptable in all but one data point, even when evaluated by MS response, indicating that the method is suitable for semi-quantitative monitoring of BSA oligomerization in parallel with UV-based quantitation.

### 2.6. Comparison of Results to an Orthogonal Detection Technique

Finally, to demonstrate that the developed method gives comparable results to orthogonal techniques, which are more established in oligomer and aggregate analysis, the results given above were compared to results obtained from SEC-RALS/LALS detection. Because the technique for quantification between the two approaches is the same (UV or RI detection), differences may arise mainly in the determined molecular weights of individual peaks—therefore, the aim of the comparison was to confirm the molecular weights of oligomeric species as determined by HRMS and that higher order aggregates form during stress testing, which could not be confirmed by HRMS.

The method was transferred to a SEC-RALS/LALS system and modified to ensure reproducibility of the results. Namely, the flow rate was increased in order to overcome the limitations of the HPLC system used. A larger diameter column was used to allow for the required increase in flow rate. Critically, sample preparation and mobile phase composition were kept constant to retain the native structure of BSA and its higher molecular weight species.

**Figure 7:**
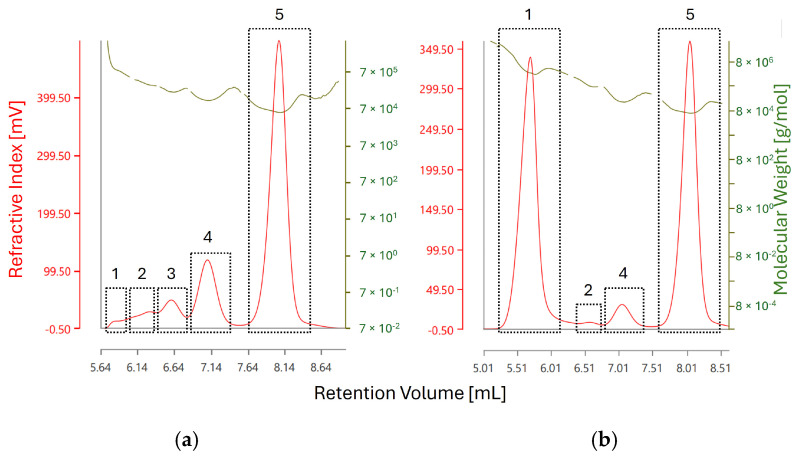
RI chromatograms of BSA (**a**) before stress testing and (**b**) after heating at 60 °C for 60 min. The red trace corresponds to the RI detector response whereas the olive-green trace corresponds to molecular weight measurement as obtained by light scattering detection. The individual RI peaks are labeled as the results given in Table 3. Peak 5 in both chromatograms corresponds to monomeric BSA, whereas peak 1 corresponds to the fraction of aggregated BSA with the highest recorded molecular weight.

In Table 3, the molecular weights of each chromatographic peak at different time points of stress testing, as determined by RALS/LALS detection, are given. Light scattering detection confirms the presence of monomeric BSA corresponding to Peak 5 in Table 3, which is in agreement with the results obtained by SEC-MS. Oligomeric species of BSA were detected between retention volumes of 6 and 7.5 mL, which in unstressed BSA range from dimers (approx. 132 kDa) to pentamers (approx. 330 kDa) according to their determined molecular weights, again confirming results obtained by SEC-MS. Critically, measurements of molecular weight obtained by RALS/LALS are not exact and become increasingly inaccurate towards lower retention volumes and at later time points of stress testing, likely due to increase in the concentration of higher molecular weight species leading to coelution—see Figure 7.

**Figure 8:**
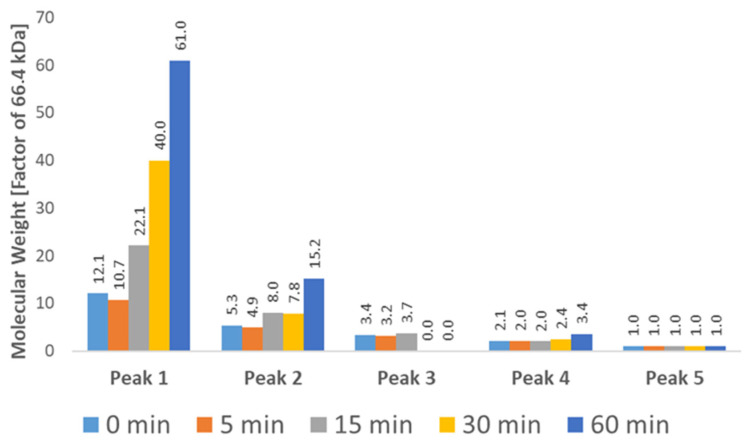
Molecular Weight (Mw) of each chromatographic peak at different time points as determined by SEC-RALS/LALS. A clear increase in molecular weight is visible for the first two peaks, which can be attributed to the growth of aggregates. The highest recorded molecular weight (4,054,362 Da) roughly corresponds to a 61-mer of BSA. The exact weight of each species is difficult to determine due to coelution, especially in the case of lower weight aggregates (Peaks 3–4).

Additionally, the presence of higher order aggregates at retention volume of approx. 5.6 mL could be confirmed by light scattering detection, corresponding to Peak 1. The molecular weight of the observed aggregates increases from 801 kDa (corresponding to a 12-mer with a theoretical weight of 792 kDa) in unstressed BSA to 4 MDa (corresponding to a 61-mer with a theoretical weight of 4026 kDa), indicating an increase in aggregate size during stress testing—see Figure 8.

The same aggregates are detected by UV and RI but do not give a measurable MS response, as previously mentioned. Taken as a whole, these results confirm the molecular weights as determined by HRMS, but also clearly demonstrate the advantages of high-resolution MS-based detection for the determination of exact molecular weights and for monitoring of each individual species where MALS detection is hindered by coelution. Additionally, the drawbacks of HRMS detection for the determination of higher-order aggregates are demonstrated. In this area, further research and technological innovation are needed to improve the mass range of modern mass spectrometers and the ionization and transmission of high molecular weight analytes.

## 3. Materials and Methods

Bovine serum albumin was purchased from Sigma Aldrich (St. Louis, MO, USA). A Milli-Q Advantage water purification system (Burlington, MA, USA) was used for preparation of deionized water for sample and mobile phase preparation. LC-MS grade methanol and ammonium acetate from Supelco^®^ Analytical Products (Sigma Aldrich) were used for mobile phase preparation. LC-MS grade acetic acid from Supelco^®^ Analytical Products and LC-MS grade 25% (*v*/*v*) ammonium hydroxide solution from Honeywell International Inc. (Charlotte, NC, USA) were used for adjusting solution pH during sample preparation.

### 3.1. Sample Preparation

Bovine serum albumin was prepared in 100 mM ammonium acetate at 20 mg/mL (150 µmol/L) prior to SEC-UV and SEC-MS analysis.

### 3.2. Mobile Phase Preparation

For mobile phase preparation, ammonium acetate was dissolved in deionized water at 50 mM, and the solution was sonicated for 5 min prior to analysis. Where stated, a volumetric mixture of water and methanol was used in place of pure water.

### 3.3. Sample Stress Testing

Prior to stress testing, solutions of BSA were transferred to 2 mL HPLC crimp vials produced by Agilent Technologies, Inc. (Santa Clara, CA, USA). The vials were sealed to prevent solvent evaporation and transferred to a dry heating block for stress testing. Each vial was heated for the prescribed period of time and transferred to a cooled autosampler for analysis.

### 3.4. Size-Exclusion Chromatography—Mass Spectrometry

An Agilent 1290 Infinity II UPLC system was used for chromatographic separation, comprising a 1290 Infinity II high-speed binary pump, 1290 Infinity II multisampler, 1290 Infinity II multi-column manager, and 1290 Infinity II DAD detector. A 10 mm flow cell was used for UV detection. The UPLC system was coupled to an Agilent 6546 qTOF mass spectrometer via the Agilent Dual Jet Stream hESI source. Initial development started on the Waters UPLC Protein BEH, 125 Å, 300 × 4.6 mm, 1.7 µm, and Agilent AdvanceBio SEC, 120 Å, 300 × 4.6 mm, 1.9 µm columns. After optimization, an AdvanceBio SEC, 200 Å, 150 × 2.1 mm, 1.9 µm PK column was purchased.

### 3.5. SEC-MS Chromatographic Conditions

Separation was carried out at an isocratic flow rate of 0.05 mL/min. The time of analysis for each run was 12 min; however, shorter run times of 10 min were used for high-throughput analysis. The column temperature was set to 35 °C, and the multisampler temperature was set to 7 °C. At these conditions, an on-column pressure of approximately 200 bar was measured. Sample injection volume was set to 5 µL, corresponding to approx. 1.5 nmol of BSA on column. UV detection was carried out from 190 nm to 640 nm, sampled in 2 nm increments with a sampling rate of 5 Hz. No reference wavelength was used during detection. UV chromatograms used for quantitation were extracted at 280 nm with a 4 nm extraction window.

### 3.6. MS Source Parameters

The flow from the UV detector was led directly to the ion source without the use of flow splitting. The source conditions were as follows: Drying Gas Temperature 200 °C, Drying Gas Flow 12 L/min, Nebulizer pressure 35 psi, Sheath Gas Temperature 50 °C, Sheath Gas Flow 6 L/min, Capillary Voltage (VCap), 5.5 kV, Nozzle Voltage 2.0 kV, Fragmentor Voltage 380 V, Skimmer Voltage 65 V, Octopole 1 RF Vpp 750 V. Spectra were acquired at 0.5 Hz.

### 3.7. Size-Exclusion Chromatography—Light Scattering Detection

An Agilent 1260 Infinity HPLC system was used for chromatographic separation, comprising of a 1260 Infinity isocratic pump, 1260 Infinity autosampler, and 1260 Infinity column manager. The HPLC system was coupled with Malvern Panalitycal OMNISEC REVEAL multi-detector system (Malvern, UK), allowing for light scattering (RALS and LALS) and differential refractive index detection.

### 3.8. SEC-RALS/LALS Chromatographic Conditions

Due to the limitations of the HPLC system used, a Tosoh Bioscience (Tokyo, Japan) TSKgel G3000SWXL, 300 × 7.8 mm, 5 um was used as the mobile phase-flow rate increased to 0.7 mL/min. The mobile phase composition, autosampler temperature, and injection volume and column temperature were left unchanged from the conditions used for SEC-MS analysis.

### 3.9. Data Processing

MassHunter Acquisition 10.0 was used for SEC-MS data acquisition. MassHunter Qualitative Analysis 10.0 was used for qualitative data processing and UV-based quantitation, and MassHunter Quantitative Analysis 10.0 was used for quantitation of oligomers using extracted ion chromatograms. For quantitation, the most MS response for the charge state with the highest abundance was determined, and MS chromatograms were extracted in a 20 Da window centered on the response maximum. Deconvolution of mass spectra and graphing of data were done in UniDec version 7.0.2 as published by Marty et al. [31]. SEC-RALS/LALS data acquisition and data processing were performed using the Malvern Panalitycal OMNISEC software v 11.40.8382.4 (13 December 2022).

### 3.10. Validation Methodology

#### 3.10.1. Linearity

Method linearity refers to the ability of an analytical method to provide test results that are directly proportional to the concentration (or amount) of analyte in a given sample, within a defined range. In the case of the determination of higher molecular weight species of BSA, which are formed in a sample during stress testing, the method should be linear in the range from the working concentration of the sample (20 mg/mL) down to the lower limit of quantification. As no reference standards for higher molecular weight species of BSA are available, the linearity of the method was evaluated by preparing several dilutions of a sample of unstressed BSA, ranging from 20 mg/mL to 0.1 mg/mL. The peak areas of monomeric and oligomeric species of BSA were determined using UV detection and plotted as a function of total BSA concentration in the sample. The correlation coefficient was determined with an acceptance criterion of R^2^ ≥ 0.99. Because of the limited dynamic range of MS-based detection as compared to UV detection, the linearity of the MS response was only qualitatively evaluated.

#### 3.10.2. Limit of Detection and Limit of Quantification

The Limit of Detection (LoD) is the lowest amount of analyte in a sample that can be detected but not necessarily quantified accurately. As per ICH Q2, the limit of detection can be defined as the lowest concentration of analyte, which still produced a response with a signal-to-noise ratio of at least 3:1. Similarly, the Limit of Quantification is the lowest amount of analyte that can be quantitatively determined with acceptable precision and accuracy and is defined as the lowest concentration of analyte, which still produced a response with a signal-to-noise ratio of at least 10:1 as per ICH Q2.

Because of the lack of reference materials, only the concentration of BSA can reasonably be determined using the described method. Therefore, LoD and LoQ of the UV response of BSA were determined by calculating the signal-to-noise ratio at the lower concentrations of the sample analyzed.

#### 3.10.3. Precision and Repeatability

In method validation (per ICH Q2(R1/R2)), precision is the measure of closeness of agreement between a series of measurements obtained from multiple sampling of the same homogeneous sample under prescribed conditions. In order to test the precision of the method, a sample of unstressed BSA was prepared at 20 mg/mL in six replicates and analyzed using UV detection. Additionally, each replicate was diluted to 2, 0.2, and 0.02 mg/mL and analyzed in order to test for method precision at different concentration levels. The relative standard deviations of the peak areas of monomeric and oligomeric BSA (as determined by UV) were calculated with an acceptance criterion of RSD ≤ 5.0%. Similarly, the precision of the MS response for monomeric BSA and each detected oligomeric species of BSA was determined by calculating the relative standard deviations of their respective peak areas at the lowest sample concentration where the species was detected, with an acceptance criterion of RSD ≤ 15.0%. The proposed precision protocol is tied to the specific formulation type that contained bio-active proteins. This is usually liquid injectable form. In such cases, the sample preparation usually contains only the dilution step. In general, the whole sample preparation process needs to be included in accurate evaluation of the precision.

As the use of the method is tied to rapid stress testing of the samples, a sample of BSA was prepared in three replicates and stressed at 60 °C for 5, 15, 30, and 60 min as per the developed stress-testing protocol. The relative standard deviations of the peak areas of monomeric, oligomeric, and aggregated BSA (as determined by UV and MS) were calculated with an acceptance criterion of RSD ≤ 15.0%.

#### 3.10.4. Stability

As the relative abundances of individual species in a solution of BSA can change during storage, the stability of the sample solution before stress testing was evaluated. The prepared solutions were stored in the autosampler at 7 °C after analysis and analyzed again after approximately 12 and 24 h of storage. The peak areas of monomeric, oligomeric, and aggregated BSA were determined using UV and MS and compared at different times of analysis. The acceptance criterion for the stability of solutions was ≤15% change in peak area between the first and each subsequent analysis.

## 4. Conclusions

A non-denaturing SEC-UV/HRMS method was successfully developed for monitoring the aggregation of bovine serum albumin (BSA) in solution. Optimization of the mobile-phase composition demonstrated that native separations can be achieved under conditions compatible with electrospray ionization without compromising chromatographic performance or sensitivity. The addition of up to 20% (*v*/*v*) methanol improved MS sensitivity while preserving the native structure of BSA. The use of ultra-performance SEC columns enabled operation at flow rates similar to direct infusion, allowing lower source temperatures and thereby reducing the risk of protein denaturation while improving ionization efficiency.

Source optimization revealed that elevated gas temperatures are the main contributor to protein unfolding, whereas their effect on ionization efficiency is relatively small. Consequently, reducing gas temperature should be prioritized during method optimization. Adjusting chromatographic parameters such as column length and inner diameter allows operation at lower flow rates, while ionization and ion-transfer voltages have minimal impact on protein unfolding but significantly improve ionization efficiency.

The developed SEC-UV/HRMS method enabled quantification of BSA monomer, as well as detection and semi-quantitative monitoring of BSA oligomers ranging from dimers to pentamers, including species that were not fully resolved or were below the UV detection limit. Higher-order aggregates showed insufficient MS response and were therefore monitored by UV detection.

The method also enables high-throughput stress testing of BSA stability. Aggregation was observed after 60 min of incubation at elevated temperature, and significant differences in oligomer formation kinetics were detected across different temperature conditions. The results show that the SEC-UV/MS method can probe early aggregation events before visible aggregates form.

Method validation showed linear UV detection for monomeric BSA from 0.02 to 20 mg/mL and for oligomeric BSA from 0.2 to 20 mg/mL, with relative standard deviations below 5%. Reliable precision was achieved down to 0.2 mg/mL, with near-acceptable precision for monomeric BSA, even at 0.02 mg/mL. Repeatability criteria were met throughout stress testing, and BSA samples remained stable for at least 24 h at 7 °C.

MS detection showed lower precision at very low concentrations but acceptable performance at the standard working concentration of 20 mg/mL, where RSD values for oligomeric species remained below 15%. The method therefore enables semi-quantitative monitoring of BSA oligomerization alongside UV-based quantification.

Comparison of molecular weights determined by HRMS with those obtained using RALS/LALS showed good agreement when oligomeric species were chromatographically resolved. HRMS provided advantages in cases of coelution, whereas orthogonal detection confirmed the formation of higher-order aggregates that could be quantified by UV or RI detection, highlighting both the strengths and limitations of HRMS-based detection.

The developed SEC-UV/HRMS procedure enables accurate quantification of studied monomer and identification, and only semiquantitative evaluation of oligomer. Such performance of described analytical procedure resolves the problem of early detection of agglomeration processes but is unable to accurately quantify oligomer development. Further development will be needed to quantitatively separate formed oligomers, which will enable their more reliable quantification as well.

## Figures and Tables

**Table 1:** Relative Peak Areas of HMWSs and monomeric BSA at different temperatures and time points, acquired using UV detection at 280 nm.

Temperature	50 °C	60 °C	70 °C
Time Point	HMWS [%]	HMWS [%]	HMWS [%]
0 min	30.49	30.52	30.33
5 min	29.98	30.23	31.75
15 min	29.86	42.52	88.35
30 min	30.04	50.53	-
60 min	30.51	56.10	-

Results where determination of BSA and HMWS content was not possible due to observed opalescence of the solution are marked with “-“.

**Table 2:** Results of method validation.

		HRMS Data						UV Data		
**Linearity**	**Parameter**	**2 M**	**3 M**	**4 M**	**5 M**	**BSA**	**HMWS**	**Agg.**	**Olig.**	**BSA**
	R2	-	-	-	-	-	-	-	0.9962	0.9983
	Slope	-	-	-	-	-	-	-	1002.6	2241.2
	Intercept	-	-	-	-	-	-	-	387.58	171.71
**LoD & LoQ**	**Parameter**	**2 M**	**3 M**	**4 M**	**5 M**	**BSA**	**HMWS**	**Agg.**	**Olig.**	**BSA**
	Lowest Concentration of Detection [mg/mL]	0.02	0.2	2.0	2.0	0.02	0.2	-	0.2	0.02
	S/N at Lowest Concentration	4	36	13	13	65	-	-	107	48
	RSD (*n* = 6) at Lowest Concentration	17.01%	18.22%	7.32%	9.03%	1.48%	0.68%	-	0.68%	5.39%
**Precision**	**Parameter**	**2 M**	**3 M**	**4 M**	**5 M**	**BSA**	**HMWS**	**Agg.**	**Olig.**	**BSA**
	RSD (*n* = 6) at 0.02 mg/mL BSA	17.01%	-	-	-	1.48%	-	-	-	5.39%
	RSD (*n* = 6) at 0.2 mg/mL BSA	5.25%	18.22%	-	-	0.30%	0.68%	-	0.68%	0.40%
	RSD (*n* = 6) at 2.0 mg/mL BSA	2.25%	4.73%	7.32%	9.03%	0.89%	1.87%	-	1.87%	0.36%
	RSD (*n* = 6) at 20 mg/mL BSA	2.11%	0.57%	5.05%	5.04%	0.84%	0.45%	-	0.45%	0.11%
**Repeatability**	**Parameter**	**2 M**	**3 M**	**4 M**	**5 M**	**BSA**	**HMWS**	**Agg.**	**Olig.**	**BSA**
	RSD (*n* = 6) at t0	1.36%	2.80%	2.90%	4.69%	1.14%	0.54%	-	0.54%	0.41%
	RSD (*n* = 3) at 60 C/5 min	0.88%	0.73%	5.03%	1.22%	0.94%	0.37%	-	0.37%	0.14%
	RSD at (*n* = 3) 60 C/15 min	2.52%	2.67%	6.51%	4.84%	0.63%	1.59%	2.97%	3.06%	0.79%
	RSD at (*n* = 3) 60 C/30 min	1.91%	5.33%	8.35%	9.57%	0.79%	0.97%	0.97%	-	0.82%
	RSD at (*n* = 3) 60 C/60 min	1.52%	9.20%	5.85%	4.89%	1.05%	1.00%	1.00%	-	0.89%
**Stability**	**Parameter**	**2 M**	**3 M**	**4 M**	**5 M**	**BSA**	**HMWS**	**Agg.**	**Olig.**	**BSA**
	Normalised Response at t0	100%	100%	100%	100%	100%	100%	100%	-	100%
	Normalised Response after 12 h	100%	95%	82% *	93%	103%	95%	98%	-	98%
	Normalised Response after 24 h	99%	93%	95%	101%	105%	96%	99%	-	99%

Note: Method was validated as per ICH Q2 (R1/R2) guidelines [29,30], where UV detection is interpreted as a quantitative test of impurities and HRMS identification is interpreted as an identification test with a much smaller list of critical performance characteristics. Additionally, because no reference materials for BSA oligomers and aggregates are available, only the total concentration of BSA in solutions is known. Consequently, RSD and S/N are provided for the lowest total concentration of BSA where each individual species could be reliably detected; this concentration is provided as “Lowest Concentration of Detection”. Because no higher-order aggregates are present in the unstressed solution used for calculation of S/N and RSD, only values for oligomeric species are provided. The relatively low normalized response of BSA tetramer (marked with “*”) is likely a consequence of measurement error, as none of the other oligomeric species show a drop in normalized response at the same timepoint. Furthermore, the tetramer response increases again after 24 h, indicating that the value measured after 12 h is a consequence of measurement error, as an increase of only tetrameric BSA is highly unlikely.

**Table 3:** Molecular Weight of each chromatographic peak as determined by SEC-MALS.

Temperature	Peak 1	Peak 2	Peak 3	Peak 4	Peak 5
0 min	800,953	350,152	225,114	140,139	66,673
5 min	710,894	323,443	210,412	135,617	64,719
15 min	1,470,740	533,882	246,353	133,363	63,253
30 min	2,660,219	521,521	-	161,158	65,859
60 min	4,054,362	1,010,628	-	229,295	67,569

Results where integration of Peak 3 was not possible due to coelution with other peaks are marked with “-“.

## Data Availability

The original contributions presented in the study are included in the article. Further inquiries can be directed to the corresponding author.

## Abbreviations

The following abbreviations are used in this manuscript:

Agg.	Aggregates
BEH	Bridged Ethylene Hybrid
BSA	Bovine serum albumin
DAD	Diode array detector
EIC	Extracted ion chromatogram
ESI	Electrospray ionization
ESI-MS	Electrospray ionization mass spectrometry
hESI	Heated electrospray ionization
HPLC	High-performance liquid chromatography
HMWS	Higher molecular weight species
HPSEC	High-performance size-exclusion chromatography
HRMS	High-resolution mass spectrometry
ICH	International Council for Harmonisation
LALS	Low-Angle Light Scattering
LC-MS	Liquid chromatography–mass spectrometry
LoD	Limit of detection
LoQ	Limit of quantification
M	Monomer (e.g., 2 M = dimer, 3 M = trimer)
MALS	Multi-angle light scattering
MS	Mass spectrometry
Mw	Molecular weight
O	Oligomer
PK	Polyether ether ketone
qTOF	Quadrupole time-of-flight
RALS	Right-Angle Light Scattering
RI	Refractive index
RSD	Relative standard deviation
Rt	Retention time
S/N	Signal-to-noise ratio
SEC	Size-exclusion chromatography
SEC-HRMS	Size-exclusion chromatography–high-resolution mass spectrometry
SEC-MALS	Size-exclusion chromatography–multi-angle light scattering
SEC-MS	Size-exclusion chromatography–mass spectrometry
SEC-RI-MALS	Size-exclusion chromatography–refractive index–multi-angle light scattering
SEC-UV	Size-exclusion chromatography with ultraviolet detection
UHPLC	Ultra-high-performance liquid chromatography
UP-SEC	Ultra-performance size-exclusion chromatography
UPLC	Ultra-performance liquid chromatography
UV	Ultraviolet
VCap	Capillary voltage
Vpp	Volts peak-to-peak
